# Consanguinity and susceptibility to infectious diseases in humans

**DOI:** 10.1098/rsbl.2009.0133

**Published:** 2009-03-18

**Authors:** Emily J. Lyons, Angela J. Frodsham, Lyna Zhang, Adrian V.S. Hill, William Amos

**Affiliations:** 1 The Wellcome Trust Centre for Human Genetics, University of Oxford, Roosevelt Drive, Oxford OX3 7BN, UK; 2 Department of Zoology, University of Cambridge, Downing Street, Cambridge CB2 3EJ, UK

**Keywords:** inbreeding, tuberculosis, human, genome scan, microsatellite

## Abstract

Studies of animal populations suggest that low genetic heterozygosity is an important risk factor for infection by a diverse range of pathogens, but relatively little research has looked to see whether similar patterns exist in humans. We have used microsatellite genome screen data for tuberculosis (TB), hepatitis and leprosy to test the hypothesis that inbreeding depression increases risk of infection. Our results indicate that inbred individuals are more common among our infected cases for TB and hepatitis, but only in populations where consanguineous marriages are common. No effect was found either for leprosy, which is thought to be oligogenic, or for hepatitis in Italy where consanguineous marriages are rare. Our results suggest that consanguinity is an important risk factor in susceptibility to infectious diseases in humans.

## 1. Introduction

Increasing numbers of papers report a link between genetic diversity and disease susceptibility, particularly in natural populations of animals (Coltman *et al.* 1999; Acevedo-Whitehouse *et al.* 2003). By implication, infection is far from random and relatively homozygous individuals may play a key role in the maintenance of pathogens in a population. Two non-exclusive mechanisms may be responsible: inbreeding depression and chance linkage between a marker and an immune-related gene experiencing balancing selection (Hansson & Westerberg 2002; Kaeuffer *et al.* 2008). Recent studies have suggested that both mechanisms may play a role and may even affect different aspects of the same disease (Acevedo-Whitehouse *et al.* 2006).

It remains unclear whether these studies of animals have any relevance to human disease because medical intervention may negate or mask any effect. In addition, with respect to inbreeding depression, most human populations are large enough to ensure that inbred individuals are likely to be extremely rare (Balloux *et al.* 2004). However, in several cultures, second-cousin marriages are actively encouraged, potentially exposing such populations to fitness differentials noted elsewhere (Jaber *et al.* 1997).

Complex pedigree analysis is commonly used to determine the degree of inbreeding in human populations. With this approach, consanguinity has been implicated in susceptibility to a number of human diseases including heart disease, multiple sclerosis, depression and asthma (Roberts 1991; Becker *et al.* 2001). By contrast, infectious diseases have received less attention, in part because of the difficulty in obtaining large numbers of deep, well-resolved pedigrees in the developing world where the major infectious diseases occur most commonly. Here, in what we believe is the first study of its kind in humans, we have revisited microsatellite genome scan linkage data for three infectious diseases in contrasting populations, to determine the extent to which genomewide heterozygosity is an important predictor of susceptibility to some diseases, particularly in populations where inbreeding is common.

## 2. Material and methods

We have reanalysed genome scan data for three important infectious diseases: tuberculosis (TB) in The Gambia (Bellamy *et al.* 2000); leprosy in India (Siddiqui *et al.* 2001); and persistent hepatitis B infection both in The Gambia and Italy (Frodsham 2000; Frodsham *et al*. 2006); these populations differing in their rates of cousin marriages from less than 1 per cent in Italy up to 43 per cent in India. All four studies were based on an affected sib-pair design, with unaffected parents acting as controls for two or more affected offspring. Sample sizes for these studies are as follows: TB in The Gambia comprising 272 autosomal markers genotyped across 263 individuals in 74 families containing 155 affected offspring, 25 affected parents, 19 unaffected offspring and 64 affected parents; hepatitis in Gambia comprising 276 autosomal markers genotyped across 280 individuals in 62 families containing 152 affected offspring, 22 affected parents, 42 unaffected offspring and 64 unaffected parents; leprosy in India comprising 390 autosomal markers genotyped in 394 individuals in 96 families containing 202 affected offspring, 51 affected parents and 141 unaffected parents; and hepatitis in Italy comprising 295 autosomal markers genotyped across 147 individuals in 32 families containing 92 affected offspring, 8 affected parents, 19 unaffected offspring and 28 unaffected parents.

Since we lack pedigrees for these populations, we use multilocus heterozygosity as a surrogate measure of the inbreeding coefficient, *F*. Specifically, we follow the method of Balloux *et al.* (2004), which estimates the degree to which heterozygosity is correlated across unlinked markers. Data from a panel of markers are repeatedly divided into randomly selected groups of approximately equal size, yielding two estimates of heterozygosity for each sample. The average correlation between these paired estimates then provides a measure of consanguinity, the argument being that when inbred individuals are absent, heterozygosity is uncorrelated, while increases in either the proportion of inbred individuals or the mean *F*-value of inbred individuals both act to create and strengthen the correlation.

The structure of the affected sib-pair datasets raises questions about how best to conduct the analysis. First, the sib-pairs are genetically non-independent. Second, given the severity of these diseases, affected parents in some or many cases may be considered to carry different forms of the disease compared with their offspring and therefore may not be comparable. We therefore conducted two parallel analyses, one based on all individuals classified only by disease status, regardless of whether they were parents or offspring, and a more conservative analysis based only on unaffected parents and one randomly selected affected offspring per family. In practice, these yield almost identical results (see below). In addition, in order to assess the average level of inbreeding in each sample, we also analysed each entire dataset after scrambling affected status.

## 3. Results

There is a strong association between consanguinity and human susceptibility to both TB and persistent hepatitis B virus infection in West Africans (figure 1). The strongest association occurs in Gambians who have a moderately high (approx. 30%) frequency of first-cousin marriages. No significant association was found for persistent hepatitis in the Italian genome scan, probably due to the low levels of consanguinity and the resulting low power of the test in this population. An association was also lacking in the leprosy dataset featuring populations from Andhra Pradesh and Tamil Nadu, where the heterozygosity–heterozygosity correlations indicate similar levels of inbreeding in both cases and controls.

**Figure 1:**
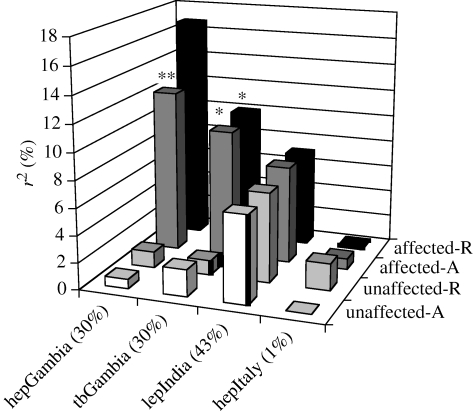
Correlations in heterozygosity among markers for affected and unaffected individuals, using data from four genome screens for infectious disease. For each disease, results are presented for both the entire dataset (suffix ‘-A’) and a conservatively restricted dataset excluding all but one affected offspring per family and all affected parents (suffix ‘-R’; see text for more details). Estimated percentage of consanguinity in each population is in brackets, obtained from http://www.consang.net (Italy and India) and Bennett *et al.* 2002 (The Gambia). Tests of significance for a difference between unaffected and affected individuals are expressed in terms of the proportion of 10 000 replicate randomizations that for unaffected individuals yielded a higher correlation than for affected individuals (^*^p<0.05, ^**^p=0.0009). hep, persistent hepatitis B; tb, tuberculosis; lep, leprosy.

When affected status is scrambled, average *r* ^2^-values are very similar in the two Gambian populations (TB=6.7%, hepatitis=6.8%) and India (6.9%), all much higher than that in Italy (0.9%). To get an idea of what these values mean, we used Monte Carlo randomizations to generate simulated datasets. Each simulation was based on the Gambian hepatitis data in terms of the number of samples, number of markers, the observed variability of each of those markers and the distribution of missing data. Genotypes were then generated for varying proportions of unrelated individuals and either the progeny of second-cousin marriages, first-cousin marriages or an equal mixture between the two (figure 2). Noticeably, the presence of appreciable numbers of first-cousin marriages appears necessary in order to account for the *r*^2^-values of 12–16% we observe in The Gambia.

**Figure 2:**
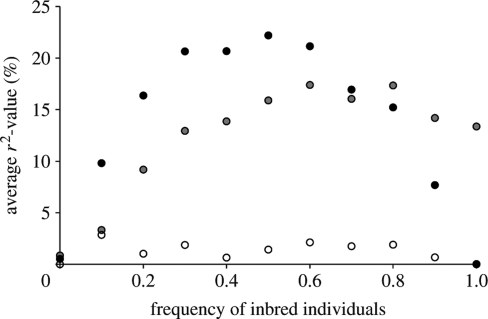
Relationship between the strength of the heterozygosity–heterozygosity correlation across markers and the proportion of inbred individuals. The proportion of inbred individuals varies between 0 and 100% in 10% intervals and data are presented for simulations where all inbred individuals are the progeny of second cousins (*F*=0.016, open circles) first cousins (*F*=0.063, black circles) and an equal mixture of the two (grey circles).

## 4. Discussion

In two of three instances where a population has high levels of consanguineous marriages, we find that affected individuals reveal significantly more evidence of inbreeding compared with unaffected controls. The exception is for leprosy in India, which may reflect its unusual genetic architecture. While most infectious diseases are probably highly polygenic, susceptibility to leprosy is strongly associated with two major effect loci, suggesting oligogenicity (Siddiqui *et al.* 2001; Mira *et al.* 2004). Alternatively, it may be that persistent, strong inbreeding in the Indian populations has led to genetic purging. Importantly, the affected sib-pair design should effectively control for biases due to population stratification that might confound a simple case–control study.

Although difficult to compare formally, the *r* ^2^-values of the heterozygosity–heterozygosity correlations suggest a stronger impact of consanguinity on hepatitis than on TB. Our simulations suggest that, in our data set, appreciable numbers of affected offspring, but many fewer unaffected parents, were born to first-cousin marriages. However, direct interpretation of *r* ^2^-values is not so straightforward. High values can arise if just one or two individuals have extreme *F*-values (whether high or low) since such data points have high leverage on the correlation. Alternatively, high values might reflect the cumulative impact of several generations of cousin marriages. By contrast, the average *r* ^2^-values of 1–2% in our Gambian controls appear in line with expectations based on the known frequencies of second-cousin marriages.

Our data comprise microsatellite genome screen data collected several years ago, before the current vogue for using single nucleotide polymorphisms (SNPs). As such, the data are similar to those collected in the numerous studies of natural populations of animals, and benefit from the high levels of polymorphism shown by individual markers, allowing smaller numbers of more widely spaced markers to be used. Inferences based on SNP data require much larger numbers of markers, which are therefore more tightly linked and, although methods are being developed for inferring individual inbreeding coefficients, these tend to suffer from a high variance of the estimate and require ‘known’ allele frequencies (Leutenegger *et al.* 2003; Carothers *et al.* 2006). Nonetheless, work is now underway to put approximately one million SNP markers across this dataset.

In conclusion, consanguinity appears significantly to increase the risk of two major infectious causes of death in humans. Rates of consanguinity are the highest in populations that are subject to the greatest burden of infectious disease mortality and many traditional human societies may have had even higher rates. Additionally, increased susceptibility to lethal infections in consanguineous individuals may have had a major impact on the evolutionary selection of pathogen resistance loci.
